# Development of a Sustainable Natural Brown Colorant from Date Seed Waste: Taguchi Optimization and Storage Stability

**DOI:** 10.3390/foods15152581

**Published:** 2026-07-23

**Authors:** Wardah S. Hasan, Javeria Mohsin, Wafa Z. Jafri, Dali V. Francis, Rema M. Amawi

**Affiliations:** 1 Mathematics and Sciences Department, Rochester Institute of Technology, Dubai 341055, United Arab Emirates; 2 Liberal Arts Department, Rochester Institute of Technology, Dubai 341055, United Arab Emirates; jm5538@rit.edu; 3 Business and Management Department, Rochester Institute of Technology, Dubai 341055, United Arab Emirates; wzj1147@rit.edu

**Keywords:** date seed waste, natural colorant, food colorant, Taguchi optimization, antioxidant activity, phenolic compounds, storage stability, sustainable food

## Abstract

The growing demand for natural food colorants has increased interest in sustainable alternatives derived from agro-industrial by-products. This study developed and characterized a food-grade natural brown colorant from date seed waste of *Phoenix dactylifera* L. (Khalas variety) by process optimization, mineral profiling, phenolic characterization and analysis of its storage stability. A food-grade aqueous extraction system was employed, and a Taguchi L9 orthogonal array was used to optimize the processing parameters affecting color development. Roasting temperature was identified as the most influential factor, contributing 85.75% of the variation in color intensity. Signal-to-noise ratio analysis determined the optimum processing conditions as 200 °C roasting temperature, 5 min roasting time, and 15 min extraction time, yielding an OD_420_ value of 0.927. FTIR analysis confirmed the presence of hydroxyl, carbonyl, aromatic, and carbohydrate-associated functional groups related to thermally generated brown pigments. Further studies demonstrated that both temperature and opaqueness of the storage bottle significantly influence colorant stability. Refrigerated storage in amber containers provided superior retention of total phenolic and flavonoid contents, in addition to a superior antioxidant activity compared with room-temperature storage in clear containers. Mineral profiling revealed significantly high levels of potassium, calcium, phosphorus, and magnesium, with the mineral composition remaining largely stable during storage. HPLC analysis identified catechin and gallic acid as the major phenolic compounds, while ellagic acid was detected exclusively in the glycerol–water extract, indicating enhanced recovery of selected phenolics through solvent modification. Overall, the developed date seed colorant combines color-forming, antioxidant and nutritional properties, while providing a sustainable approach for the valorization of date-processing by-products. The findings highlight the potential application of date seed colorant as a clean-label alternative to synthetic brown colorants and support the development of sustainable food ingredients aligned with sustainable development goals.

## 1. Introduction

Consumer demand for clean-label foods has accelerated the replacement of synthetic additives with naturally derived ingredients [1]. Among these, food colorants play a critical role in consumer acceptance, product identity, and perceived quality. Although synthetic brown colorants are widely used in beverages, bakery products, confectionery products, and sauces [2], concerns regarding their safety, regulatory restrictions, and consumer perception have stimulated interest in natural alternatives [3]. Consequently, the development of stable, sustainable, and clean-label natural colorants from agricultural by-products has become an important area of food research [4].

Date palm (*Phoenix dactylifera* L.) is one of the most economically important crops in arid and semi-arid regions, particularly in the Middle East and North Africa [5]. The United Arab Emirates is among the leading producers of dates, generating substantial quantities of date-processing residues annually. Date seeds account for approximately 10–15% of the total fruit weight and are often discarded or utilized as low-value animal feed despite being rich in dietary fiber, minerals, phenolic compounds, and antioxidant constituents [6]. Despite this rich chemical composition, date seeds remain an underutilized by-product, creating an opportunity to develop value-added food ingredients that support both waste reduction and sustainable food production. The valorization of date seed waste aligns with circular economy principles by transforming an underutilized by-product into a value-added food ingredient while reducing environmental burdens associated with agro-industrial waste [7]. Such approaches also contribute to the United Nations Sustainable Development Goals (SDGs), particularly SDG 12 (Responsible Consumption and Production) by converting an underutilized agro-industrial by-product into a value-added food ingredient, SDG 9 (Industry, Innovation and Infrastructure), through the development of an optimized process for producing a sustainable natural food colorant; and SDG 13 (Climate Action), by promoting waste valorization and reducing the environmental burden associated with date seed disposal. All these aspects highlight the potential of the present research to support sustainable food production and the future development of commercially viable natural colorants.

Previous studies have demonstrated that date seeds contain appreciable amounts of phenolic acids, flavonoids, tannins, and other bioactive compounds that contribute to their antioxidant potential [8]. In addition to their nutritional value, date seeds have attracted attention as ingredients for functional foods [9], coffee substitutes [10], and nutraceutical applications [11]. Roasting is a critical processing step that enhances aroma, flavor and color development, in addition to its extractability. Thermal treatment promotes Maillard reactions, caramelization [12], and the formation of melanoidins, resulting in the generation of brown-colored compounds and antioxidant-active products [13]. These characteristics suggest that roasted date seeds may serve as a promising source of natural brown pigments for food applications. Unlike many plant-derived natural pigments that exhibit limited stability during processing and storage, thermally generated brown pigments produced through controlled roasting may offer improved color stability while simultaneously retaining antioxidant properties. This makes roasted date seeds an attractive candidate for the development of clean-label brown food colorants.

Despite growing interest in date seed utilization, research has primarily focused on coffee substitutes, antioxidant-rich extracts, and functional food ingredients, while the development of natural brown colorants remains largely unexplored. More importantly, previous studies have rarely integrated process optimization with storage stability, physicochemical characterization, and phenolic profiling to evaluate the suitability of date seed-derived colorants for practical food applications. In particular, the application of Taguchi-based optimization for the production of the date seed-derived brown colorant, together with evaluation of storage stability under different packaging and temperature conditions, has not been comprehensively explored. Furthermore, information regarding the retention of phytochemicals, antioxidant activity, mineral composition, and individual phenolic compounds during storage remains insufficient. To the best of our knowledge, this is the first study to integrate Taguchi-based process optimization, storage stability evaluation under different packaging conditions, FTIR characterization, mineral profiling, antioxidant assessment, and HPLC-based phenolic profiling for the development of a sustainable natural brown colorant from date seed waste.

Based on these identified research gaps, this study was undertaken to develop a sustainable natural brown colorant from date seed waste and evaluate its potential for clean-label food applications. Specifically, the objectives were to: (1) optimize the production of a natural brown colorant from date seed waste using Taguchi experimental design; (2) characterize the functional groups associated with color development using FTIR spectroscopy; (3) evaluate the effects of storage temperature and packaging conditions on color stability, phytochemical content, antioxidant activity, pH, and mineral composition; and (4) determine the phenolic profile of the developed colorant using HPLC. In addition to identifying the optimum conditions for aqueous extraction, the Taguchi experimental design establishes a systematic and transferable optimization framework that can be readily adapted in future studies to optimize extraction using other food-grade solvent systems. The findings provide a sustainable strategy for converting date seed waste into a value-added natural food colorant with potential applications in clean-label food formulations while supporting circular economy principles and sustainable food systems.

## 2. Materials and Methods

### 2.1. Preparation of Date Seed Powder

Date seeds of the Khalas variety (*Phoenix dactylifera* L.) were collected from a local farm in the Al Ain, United Arab Emirates (UAE). The collected seeds were thoroughly washed with distilled water to remove adhering fruit residues and impurities. The cleaned seeds were then dried in a hot air oven at 60 °C for 3–4 days until complete removal of moisture. The dried seeds were subsequently ground into fine powder using a basic laboratory grinder and stored in sealed containers for further use in the roasting and extraction experiments.

### 2.2. Preliminary Screening Experiment for Selection of Roasting Temperature

A preliminary screening experiment was conducted prior to Taguchi optimization to identify a suitable roasting temperature range for date seed colorant extraction. Date seed powder was initially roasted in a hot air oven at different temperatures ranging from 70 °C to 230 °C for 3 min under fixed roasting conditions. Subsequently, the color intensity of the extracts was evaluated visually and by measuring absorbance at 420 nm using a UV–Visible spectrophotometer (UV-1900i, Shimadzu Corporation, Kyoto, Japan). Since the primary objective of this study was to develop a natural brown colorant, the preliminary screening focused on color development to identify the most appropriate roasting temperature range for the subsequent Taguchi optimization. Roasting is the most critical processing step governing the formation of thermally generated brown pigments; therefore, color intensity was considered the most appropriate selection criterion at this stage. Following optimization, the selected extract was comprehensively characterized for its physicochemical, phytochemical, antioxidant, mineral, and phenolic properties.

Based on the preliminary screening experiment, roasting temperatures of 90, 110, and 200 °C were selected for further optimization using the Taguchi L9 experimental design.

### 2.3. Experimental Design and Statistical Analysis Using Taguchi Orthogonal Array

A Taguchi orthogonal array design was used to optimize the extraction conditions for obtaining a natural brown colorant from date seed powder. The Taguchi method was selected for its ability to evaluate multiple processing factors with a reduced number of experimental runs.

Three processing variables were selected for optimization: roasting temperature, roasting time, and extraction time. The roasting temperature range was selected based on the preliminary screening experiment, while roasting time and extraction time were included due to their possible influence on color development and extraction efficiency [14]. Each factor was studied at three levels using an L9 orthogonal array generated through Minitab Statistical Software (Version 21, Minitab Inc., State College, PA, USA). The same software was used for statistical evaluation, including signal-to-noise (S/N) ratio analysis, analysis of variance (ANOVA) regression analysis, and contour plot generation. The selected factors and levels are presented in Table 1, where T represents roasting temperature, Rt represents roasting time, and Et represents extraction time.

The experimental combinations generated through the Taguchi L9 orthogonal array are presented in Table 2.

For each experimental run, roasted date seed powder was extracted in distilled water at room temperature (25 ± 2 °C) for the extraction time specified in the Taguchi experimental design (5, 10, or 15 min) using a magnetic stirrer operated at 50 rpm. The extracts were then filtered, and the color intensity was measured as absorbance at 420 nm (OD_420_) using a UV–visible spectrophotometer.

The experimental combinations designed through the Taguchi L9 orthogonal array are shown in Table 1. For each experimental run, roasted date seed powder was subjected to extraction under the specified conditions, and the color intensity of the obtained extract was measured as absorbance at 420 nm (OD_420_) using a UV–visible spectrophotometer.

The optimization criterion selected for the Taguchi analysis was the “larger-is-better” signal-to-noise (S/N) ratio, since higher absorbance values indicated greater color extraction efficiency. The S/N ratio was calculated according to the following equation:

(1) $$S/N=-10{log}_{10}\left(\frac{1}{n}\sum \frac{1}{Y^{2}}\right)$$

where Y represents the observed response value and n denotes the number of experimental observations [15].

Main effect plots and response tables for S/N ratios were generated to determine the most influential extraction parameters and their optimal levels. Analysis of variance (ANOVA) was further performed to evaluate the statistical significance and percentage contribution of each factor toward color extraction efficiency.

Regression analysis and contour plots were used to study the effect of processing variables on color extraction and to develop a predictive equation for the extraction process. The predicted response under optimum conditions was calculated using the Taguchi prediction equation:

(2) Ŷ = Tm + Σ(Ti − Tm)

where Ŷ represents the predicted response, Tm is the overall mean response, and Ti represents the mean response at the optimum level of each factor.

The predicted values obtained from the regression model were compared with the experimental values, and percentage error was calculated to determine the accuracy of the model [16]. Confirmation experiments were further performed under the predicted optimum conditions to validate the optimization results. All experiments were performed in triplicate, and the results were expressed as mean ± standard deviation.

### 2.4. Fourier Transform Infrared Spectroscopy (FTIR) Analysis

Fourier Transform Infrared Spectroscopy (FTIR) analysis was carried out to identify the major functional groups present in the date seed powder and the extracted color compounds. The FTIR spectrum was recorded using a Shimadzu FTIR spectrophotometer equipped with an Attenuated Total Reflectance (ATR) accessory within the wavelength range of 4000–500 cm^−1^. The obtained spectra were analyzed to identify the major organic and inorganic functional groups associated with the chemical components present in the roasted date seed powder and extracted colorant [17].

### 2.5. Storage Stability Study

The optimized extraction conditions obtained from the Taguchi analysis were further used for characterization and storage stability studies of the date seed colorant extract. The extract prepared under the optimized conditions was transferred into clear and amber glass bottles and stored under room temperature and refrigerated conditions for seven days to evaluate the effect of storage temperature and light exposure on extract stability.

Four storage conditions were evaluated: clear bottle at room temperature, amber bottle at room temperature, clear bottle under refrigeration (5 °C), and amber bottle under refrigeration (5 °C). Samples were collected at Day 0, Day 2, Day 5, and Day 7 for analysis.

The stored samples were analyzed for changes in color intensity (OD_420_), pH, antioxidant activity, and phytochemical stability during storage. OD_420_ measurements were carried out using a UV–visible spectrophotometer. The aqueous extract was stored directly without removal of the extraction solvent. The seven-day storage period was designed as a preliminary investigation to evaluate the influence of packaging material and storage temperature on the initial stability of the developed colorant under different storage conditions [18].

### 2.6. Determination of Total Phenolic and Total Flavonoid Content

Total phenolic content (TPC) and total flavonoid content (TFC) were determined using colorimetric methods with slight modifications from previously reported protocols. For TPC analysis, the extract was mixed with Folin–Ciocalteu reagent and incubated at room temperature. Sodium hydroxide solution was then added, and the reaction mixture was incubated in the dark before measuring absorbance at 750 nm using a UV–visible spectrophotometer (Shimadzu). Gallic acid was used as the standard, and the results were expressed as mg gallic acid equivalents (GAE)/g dry weight [19].

For TFC analysis, the extract was sequentially mixed with sodium nitrite, aluminum chloride, and sodium hydroxide solutions. The absorbance was measured at 510 nm using a UV–visible spectrophotometer. Quercetin was used as the reference standard, and the results were expressed as mg quercetin equivalents (QE)/g dry weight [19].

### 2.7. Determination of Antioxidant Activity

The antioxidant activity of the date seed colorant extracts was evaluated using DPPH (2,2-diphenyl-1-picrylhydrazyl radical) and ABTS (2,2′-azino-bis (3-ethylbenzothiazoline-6-sulfonic acid) radical scavenging assays with slight modifications from previously reported methods. For the DPPH assay, the extract was mixed with DPPH solution and incubated in the dark before measuring absorbance at 517 nm. For the ABTS assay, the extract was reacted with the ABTS radical solution and incubated in the dark prior to absorbance measurement at 734 nm. Trolox was used as the reference standard for both assays, and antioxidant capacities were calculated from the respective calibration curves and expressed as mg Trolox equivalent antioxidant capacity per gram dry weight (mg TEAC g^−1^ DW) [20].

### 2.8. pH Measurement

The pH of the date seed colorant extracts was measured using a calibrated digital pH meter. Prior to analysis, the pH meter was calibrated using standard buffer solutions. The pH of each sample was recorded at different storage intervals under room temperature (RT) and refrigerated (5 °C) conditions using both clear and amber containers.

### 2.9. ICP-MS Mineral Analysis

Mineral profiling of the optimized date seed colorant extract was conducted using inductively coupled plasma mass spectrometry (ICP-MS, Agilent 7800 ICP-MS, Agilent Technologies, Santa Clara, CA, USA). Prior to analysis, the aqueous extract was acidified with nitric acid (HNO_3_) and analyzed for the determination of macro- and microelements, including K, Ca, P, Mg, Na, Fe, Zn, Cu, and Mn [21].

### 2.10. HPLC Analysis of Phenolic Compounds

High-performance liquid chromatography (HPLC) was used to identify and quantify individual phenolic compounds in the date seed colorant extracts. Since the colorant is intended for edible applications, distilled water was used as the main extraction solvent throughout the optimization and storage studies. Following identification of the optimum processing conditions using the Taguchi design, an additional glycerol–water (1:1) extract was prepared using the same optimized roasting and extraction parameters to evaluate whether the incorporation of a food-grade co-solvent influences the extraction of individual phenolic compounds. For both solvents, 5 g of dried sample was extracted in 50 mL of solvent. The extracts were filtered prior to HPLC analysis, which was performed using a Shimadzu LCMS-8060 triple quadrupole mass spectrometer coupled with a Nexera X2 LC-30AD UHPLC system equipped with a Kromasil C18 column (250 × 4.6 mm, 5 µm). Separation was carried out using a 0.1% orthophosphoric acid–methanol gradient mobile phase at a flow rate of 1.0 mL/min with detection at 280 nm. Phenolic compounds were identified by comparing their retention times with authentic standards, and the quantified values were expressed as µg/g dry weight (DW) [22].

### 2.11. Statistical Analysis

All experiments were conducted in triplicate, and the results were expressed as mean ± standard error (SE). Statistical analysis was performed using Minitab Statistical Software Version 21 (Minitab Inc., State College, PA, USA).

For the Taguchi optimization study, signal-to-noise (S/N) ratio analysis using the larger-is-better criterion was used to identify the optimum extraction conditions. Analysis of variance (ANOVA), regression was performed to determine the significance and percentage contribution of each processing factor. Regression analysis and contour plots were used to evaluate the relationship between processing variables and color intensity.

For physicochemical, phytochemical, antioxidant, mineral, and HPLC analyses, one-way ANOVA was performed, followed by Tukey’s post hoc test to determine significant differences among treatments at *p* ≤ 0.05.

## 3. Results

### 3.1. Preparation of Date Seed Powder

The collected Khalas date seeds were successfully cleaned, dried, and processed into fine powder for subsequent roasting and extraction experiments. Drying was done at 60 °C for 3–4 days to effectively remove residual moisture from the seeds, facilitating uniform grinding and improving powder consistency. The obtained date seed powder exhibited a light brown appearance prior to roasting and was suitable for further thermal processing and color extraction studies.

### 3.2. Preliminary Screening Experiment for Selection of Roasting Temperature

The preliminary screening experiment demonstrated a progressive increase in brown color development with increasing the roasting temperature (Table 3). The OD_420_ value increased from 0.12 at 70 °C to 1.21 at 230 °C. Samples roasted at 70 °C exhibited very light brown coloration with minimal pigment formation, whereas roasting at 90 °C increased the OD_420_ value to 0.35, indicating the initiation of brown color development.

Further increases in the roasting temperature resulted in gradual enhancement of color intensity. The OD_420_ values reached 0.51, 0.74, and 0.96 at 110 °C, 150 °C, and 200 °C, respectively. The highest absorbance value was observed at 230 °C (OD_420_ = 1.21); however, visual examination revealed excessive darkening and burnt characteristics. Based on the absorbance values and visual observations, roasting temperatures of 90 °C, 110 °C, and 200 °C were selected for subsequent Taguchi optimization studies.

### 3.3. Optimization of Date Seed Colorant Extraction Using Taguchi Design

The Taguchi L9 orthogonal array was successfully used to optimize the extraction conditions for obtaining a natural brown colorant from date seed powder. The experimental design and corresponding response values are presented in Table 4. The OD_420_ values obtained from the different experimental combinations ranged from 0.44 to 0.915, showing that the selected processing conditions strongly affected color extraction efficiency.

Among the experimental runs, the lowest OD_420_ value (0.44) was observed in Run 1 (90 °C, 1 min roasting, 5 min extraction), whereas the highest OD_420_ value (0.915) was recorded in Run 9 (200 °C, 5 min roasting, 10 min extraction) (Table 4). At a roasting temperature of 90 °C, OD_420_ values increased from 0.44 to 0.58 as roasting time increased from 1 to 5 min. Similarly, at 110 °C, OD_420_ values increased from 0.525 to 0.67 with increasing roasting time. The highest absorbance values were consistently observed at 200 °C, where OD_420_ ranged from 0.775 to 0.915. Correspondingly, S/N ratio values improved from −7.13 in Run 1 to −0.77 in Run 9, indicating enhanced color extraction performance under higher roasting conditions.

The response table for S/N ratios is presented in Table 5. Roasting temperature exhibited the highest delta value at 4.438, followed by a delta value of 1.987 for roasting time. Moreover, extraction time showed the lowest delta value of 0.404. Based on the delta values, the parameters were ranked as roasting temperature (Rank 1), roasting time (Rank 2), and extraction time (Rank 3). The mean S/N ratio increased substantially from −5.820 at Level 1 (90 °C) to −1.382 at Level 3 (200 °C), indicating a strong effect of roasting temperature on OD_420_ values. Roasting time also showed a progressive increase in S/N ratio from −4.981 at Level 1 to −2.994 at Level 3. In contrast, extraction time produced only minor variation in S/N ratios across the tested levels.

**Figure 1:**
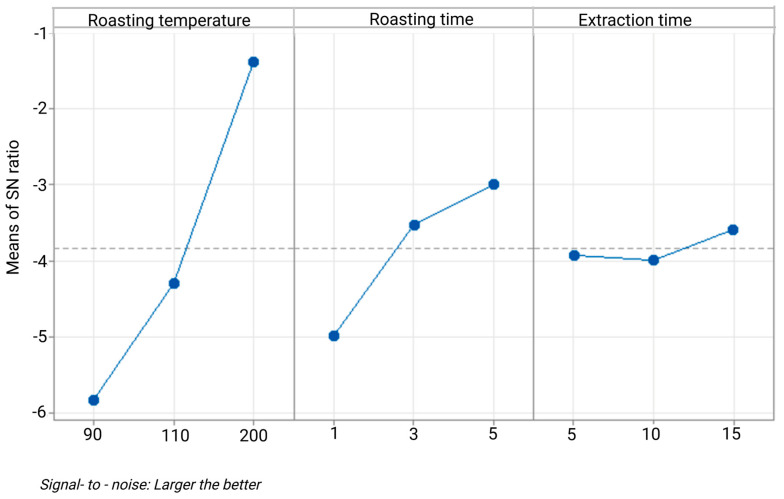
Main effect plot showing the influence of roasting temperature, roasting time, and extraction time on the signal-to-noise (S/N) ratio for date seed colorant extraction.

The main effects plot (Figure 1) further illustrated the influence of the selected factors on color extraction efficiency. A pronounced increase in S/N ratio was observed with increasing the roasting temperature, whereas the roasting time produced a moderate increase. Extraction time displayed comparatively smaller changes in S/N ratio throughout the studied range. Based on the S/N ratio analysis using the larger-is-better criterion, the optimum parameter combination was identified as 200 °C roasting temperature, 5 min roasting time, and 15 min extraction time (T_3_Rt_3_Et_3_).

**Figure 2:**
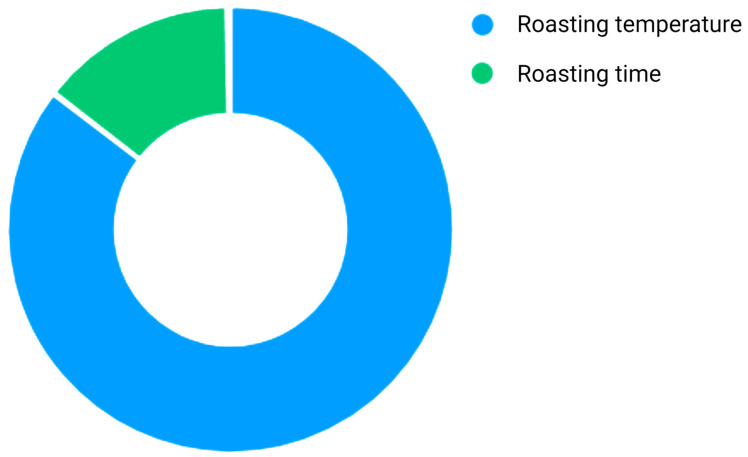
Percentage contribution of roasting temperature, roasting time, and extraction time toward date seed colorant extraction efficiency based on ANOVA analysis.

Analysis of variance (ANOVA) identified roasting temperature as the dominant factor affecting date seed colorant extraction efficiency. The percentage contribution analysis (Figure 2) showed that roasting temperature accounted for 85.75% (*p* ≤ 0.001) of the total variation in OD_420_ values, whereas roasting time contributed 14.24% (*p* ≤ 0.002). Extraction time showed a negligible contribution of only 0.02%. These results indicate that the majority of the observed variation in color intensity was associated with changes in roasting temperature.

**Figure 3:**
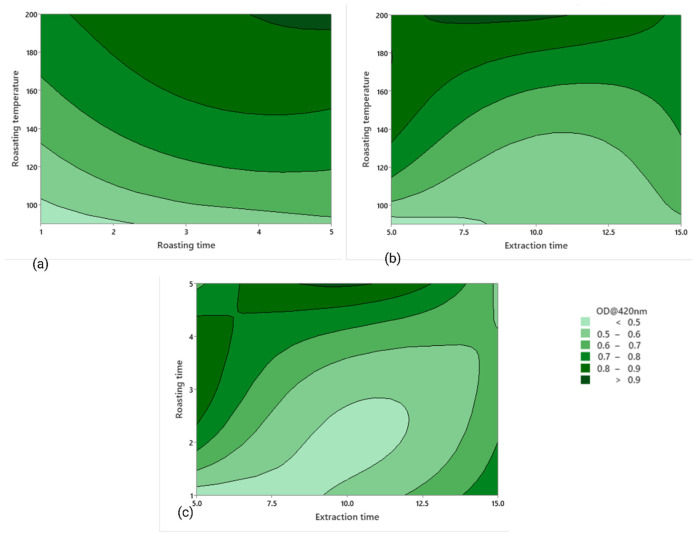
Contour plots showing the combined effect of (**a**) roasting temperature and roasting time, (**b**) roasting temperature and extraction time, and (**c**) roasting time and extraction time on OD_420_ values during date seed colorant extraction.

Contour plots illustrating the combined effects of the processing parameters on OD_420_ values are shown in Figure 3. A progressive increase in OD_420_ values towards the upper-right region was demonstrated in the contour plot between roasting temperature and roasting time (Figure 3a), corresponding to higher roasting temperatures and longer roasting times. Similarly, the contour plot between roasting temperature and extraction time (Figure 3b) showed increasing OD_420_ values with increasing roasting temperature across all extraction times. The contour plot between roasting time and extraction time (Figure 3c) revealed a gradual improvement in OD_420_ values with increasing roasting time, while extraction time exhibited comparatively smaller changes throughout the studied range.

Regression analysis was performed to develop a predictive model for OD_420_ values. The regression equation obtained was:

(3) OD_420_ = 0.1543 + 0.002967(T) + 0.03542(Rt) + 0.00050(Et)

The regression model showed a satisfactory prediction accuracy between the predicted and the experimental values resulting in an R^2^ value of 97.88. The higher regression coefficient observed for roasting temperatures further confirmed its dominant effect on brown color formation compared to extraction time.

Confirmation experiments were conducted under the predicted optimum conditions of 200 °C roasting temperature, 5 min roasting time, and 15 min extraction time to validate the optimization study (Table 6). These conditions were selected based on the best levels of each parameter obtained from the Taguchi S/N ratio analysis, where the highest mean S/N ratios were observed. Under these optimized conditions, the experimental OD_420_ value obtained was 0.927, which was in close agreement with the predicted values obtained from both the Taguchi (0.921) and regression (0.932) models. The percentage error for the confirmation experiment was less than 5%, confirming the suitability and reliability of both prediction models for date seed colorant extraction.

The Taguchi optimization identified the optimum processing conditions for date seed colorant extraction as 200 °C roasting temperature, 5 min roasting time, and 15 min extraction time, yielding an OD_420_ value of 0.927. The results consistently showed that higher roasting temperatures and longer roasting times produced greater color intensity, whereas extraction time had a comparatively smaller effect on the response.

### 3.4. FTIR Analysis of Date Seed Powder

**Figure 4:**
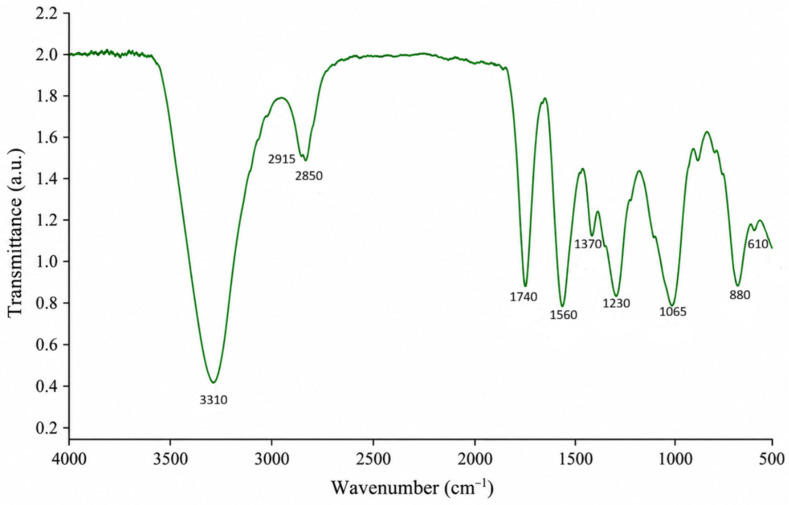
FTIR spectrum of the date seed powder.

The FTIR spectrum of roasted date seed powder is presented in Figure 4. Several absorption bands were observed across the scanned wavenumber range. A broad absorption band was detected around 3310 cm^−1^, while additional peaks were observed at approximately 2915 and 2850 cm^−1^. Distinct absorption bands were also recorded at 1740 cm^−1^ and 1560 cm^−1^. Further peaks were observed in the fingerprint region at approximately 1370, 1230, and 1065 cm^−1^. Lower-intensity bands were detected at around 880 and 610 cm^−1^ (Figure 4). These results indicate the presence of multiple functional groups within the roasted date seed powder.

### 3.5. Storage Stability Study of Optimized Date Seed Colorant Extract

**Figure 5:**
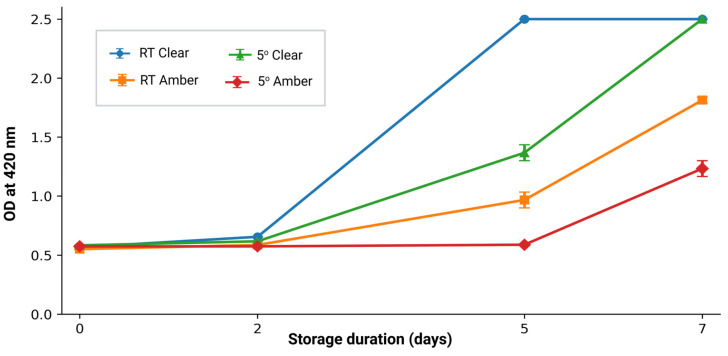
Effect of storage duration and storage conditions on OD_420_ values of optimized date seed colorant extract under different storage conditions.

The storage stability study showed that storage duration and storage conditions significantly influenced the OD_420_ values of the optimized date seed colorant extract (Figure 5). Significant differences (*p* < 0.05) were observed among treatments during the storage period.

Samples stored in clear bottles showed a greater increase in OD_420_ values compared to amber bottles. The clear bottle samples stored at room temperature exhibited the highest increase, where OD_420_ values increased from 0.567 ± 0.008 at Day 0 to 2.500 ± 0.000 by Day 5 and remained unchanged until Day 7. Clear bottle samples at refrigerated conditions also demonstrated a marked increase in OD_420_ values, increasing from 0.583 ± 0.002 at Day 0 to 2.500 ± 0.000 at Day 7.

In contrast, amber bottle samples displayed comparatively lower increases in OD_420_ values during storage. The OD_420_ value of the amber bottle samples stored at room temperature increased from 0.551 ± 0.006 at Day 0 to 1.813 ± 0.026 at Day 7, whereas amber bottle samples stored at refrigerated conditions exhibited the lowest increase, rising only from 0.574 ± 0.002 at Day 0 to 1.233 ± 0.067 at Day 7.

The results indicate that clear bottle storage promoted greater color intensity changes during storage, whereas amber bottle storage reduced the increase in OD_420_ values. Refrigerated amber bottle storage showed comparatively better color stability among the tested storage conditions.

### 3.6. Effect of Storage Conditions on Total Phenolic and Total Flavonoid Content

**Figure 6:**
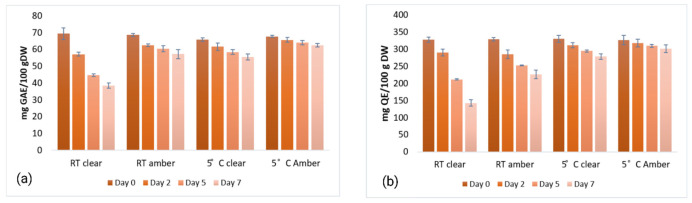
Effect of storage duration and storage conditions on (**a**) total phenolic content (TPC) and (**b**) total flavonoid content (TFC) of optimized date seed colorant extract during storage.

The effect of storage conditions on total phenolic content (TPC) and total flavonoid content (TFC) of the optimized date seed colorant extract is presented in Figure 6a and Figure 6b, respectively. Both storage duration and storage conditions significantly influenced TPC and TFC values throughout the storage period (*p* < 0.05).

TPC decreased progressively under all storage conditions during the 7-day storage period. The greatest reduction was observed in room-temperature clear bottle samples, where TPC decreased significantly (*p* < 0.05) from 69.38 mg GAE/100 g DW at Day 0 to 38.46 mg GAE/100 g DW at Day 7. Amber bottle samples stored at room-temperature exhibited a comparatively lower decline, decreasing from 68.59 to 57.20 mg GAE/100 g DW. Refrigerated samples improved phenolic retention, with refrigerated amber bottle samples showing the highest stability and decreasing only from 67.77 to 62.44 mg GAE/100 g DW during storage (Figure 6a).

A similar trend was observed for TFC (Figure 6b). Flavonoid content decreased significantly (*p* < 0.05) under all storage conditions over time. The largest reduction was recorded in clear bottle samples stored at room-temperature, where TFC declined from 327.64 mg QE/100 g DW at Day 0 to 142.81 mg QE/100 g DW at Day 7. Amber bottle samples stored at room-temperature decreased from 329.17 to 226.53 mg QE/100 g DW during storage. Refrigerated samples retained higher flavonoid levels throughout the storage period, with refrigerated amber bottle samples showing the greatest stability and maintaining TFC values above 300 mg QE/100 g DW even after 7 days of storage. The refrigerated amber bottle storage provided the highest retention of both phenolic and flavonoid compounds, whereas clear bottle stored at room-temperature resulted in the greatest losses during storage.

### 3.7. Antioxidant Activity During Storage

#### 3.7.1. ABTS Antioxidant Activity

**Figure 7:**
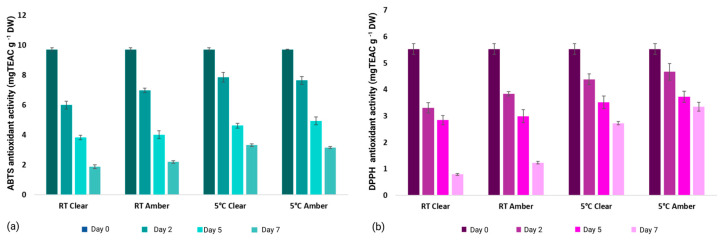
Changes in antioxidant activity of date seed colorant extracts during storage as determined by (**a**) ABTS and (**b**) DPPH assays.

The ABTS antioxidant activity of the date seed colorant extracts decreased significantly (*p* ≤ 0.05) during storage under all conditions (Figure 7a). The initial ABTS value for all samples was 9.71 ± 0.12 mg TEAC g^−1^ DW at Day 0. Under room-temperature storage, antioxidant activity decreased to 5.99 ± 0.27 and 6.97 ± 0.15 mg TEAC g^−1^ DW in RT clear and RT amber samples, respectively, after 2 days. By Day 7, the values further declined to 1.88 ± 0.13 and 2.20 ± 0.08 mg TEAC g^−1^ DW, respectively. Samples stored at 5 °C retained significantly higher antioxidant activity throughout the storage period, with values of 3.32 ± 0.09 mg TEAC g^−1^ DW (5 °C clear) and 3.16 ± 0.07 mg TEAC g^−1^ DW (5 °C amber) after 7 days. Refrigerated samples preserved approximately 33–34% of the initial ABTS antioxidant activity, whereas samples stored at room-temperature retained only 19–23%.

#### 3.7.2. DPPH Antioxidant Activity

A similar trend was observed for DPPH antioxidant activity (*p* ≤ 0.05) (Figure 7b). The initial DPPH value was 5.53 ± 0.20 mg TEAC g^−1^ DW for all samples. Samples stored in clear and amber bottles at room-temperature demonstrated a rapid decline in the DPPH antioxidant activity decreasing to 3.31 ± 0.20 mg TEAC g^−1^ DW (25 °C clear) and to 3.83 ± 0.08 mg TEAC g^−1^ DW (25 °C amber) respectively by day 2, and a further decline to 0.80 ± 0.03 and 1.24 ± 0.05 mg TEAC g^−1^ DW by day 7. Samples stored at refrigerated conditions revealed significantly greater antioxidant retention, maintaining 2.72 ± 0.06 mg TEAC g^−1^ DW (5 °C clear) and 3.35 ± 0.17 mg TEAC g^−1^ DW (5 °C amber) after 7 days. Among all treatments, the 5 °C amber samples exhibited the highest DPPH antioxidant retention, while RT clear samples showed the greatest loss of activity.

The antioxidant retention was higher in samples stored at refrigerated conditions than in those stored at room-temperature. Among all treatments, amber bottles stored at refrigerated conditions showed the highest retention of DPPH activity, while clear bottles stored at room-temperature exhibited the greatest loss of antioxidant activity.

### 3.8. Changes in pH During Storage

**Figure 8:**
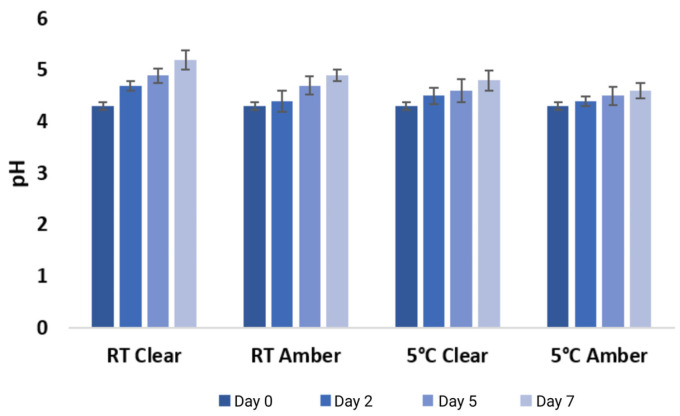

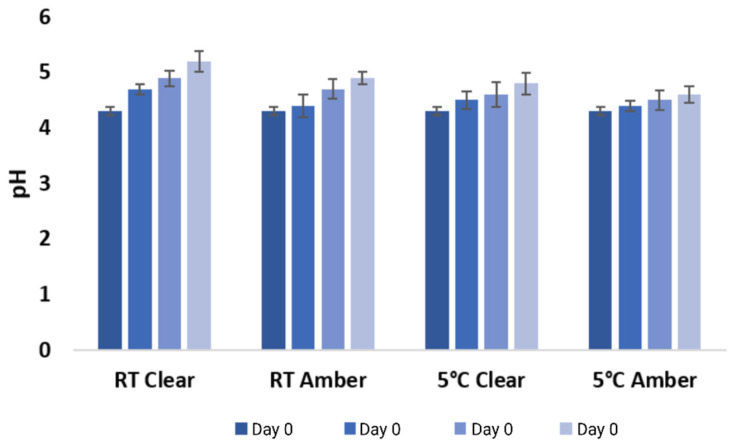
Changes in pH of date seed colorant extracts during storage under different conditions.

The pH of the date seed colorant extracts increased significantly (*p* ≤ 0.05) during storage under all conditions (Figure 8). The initial pH of all samples was 4.30 ± 0.07 at Day 0. The pH increased to 4.70 ± 0.09 and 4.40 ± 0.21 in samples stored at room temperature in both clear and amber bottles, respectively, after 2 days. By Day 7, the pH further increased to 5.20 ± 0.18 in samples stored at room temperature in clear bottles, and 4.90 ± 0.11 in samples stored at room temperature in amber bottles.

Samples stored at refrigerated condition demonstrated a slower increase in pH throughout the storage period. The pH values reached 4.50 ± 0.16 and 4.40 ± 0.09 after 2 days in 5 °C clear and 5 °C amber samples, respectively. After 7 days, the pH increased to 4.80 ± 0.20 in 5 °C clear samples and 4.60 ± 0.15 in 5 °C amber samples. Among all storage conditions, samples stored at room temperate in clear bottles exhibited the highest pH increase, whereas samples stored in refrigerated conditions, in amber bottles showed the smallest increase during storage.

### 3.9. Mineral Composition During Storage

The mineral composition of the date seed colorant extracts is presented in Table 7. Analysis of the aqueous extract revealed the presence of both macro- and microelements, including potassium (K), calcium (Ca), phosphorus (P), magnesium (Mg), sodium (Na), iron (Fe), zinc (Zn), copper (Cu), and manganese (Mn). Potassium was the most abundant mineral, with concentrations ranging from 109.2–118.5 mg/100 g DW, followed by calcium (30.9–33.1 mg/100 g DW), phosphorus (26.7–28.5 mg/100 g DW), magnesium (16.9–18.1 mg/100 g DW), and sodium (8.8–9.9 mg/100 g DW). Among the micronutrients, iron ranged from 2.1–3.1 mg/100 g DW, while zinc, copper, and manganese were present at lower concentrations ranging from 0.39–0.49, 0.10–0.18, and 0.08–0.11 mg/100 g DW, respectively. The concentrations of most minerals remained relatively stable throughout the 7-day storage period, although a slight reduction in iron concentration was observed in some treatments, particularly stored at room-temperature. Despite this slight reduction, the overall mineral composition remained largely preserved, indicating that the nutritional mineral value of the date seed colorant was maintained throughout storage.

### 3.10. HPLC Phenolic Profile

The HPLC phenolic profile of the date seed colorant extracts prepared using aqueous and glycerol–water extraction systems is presented in Table 8. Catechin was detected in both extraction systems at comparable concentrations, with values of 11.35 ± 0.9 µg/g DW in the aqueous extract and 11.10 ± 1.1 µg/g DW in the glycerol–water extract.

Gallic acid was also identified in both extracts, with slightly higher concentrations in the glycerol–water extract (5.25 ± 0.8 µg/g DW) compared with the aqueous extract (4.50 ± 0.5 µg/g DW). In contrast, ellagic acid was detected only in the glycerol–water extract, where it was present at a concentration of 7.80 ± 0.3 µg/g DW, while it was not detected in the aqueous extract.

The results demonstrate differences in phenolic composition between the two extraction systems, with the glycerol–water extract exhibiting a broader phenolic profile than the aqueous extract.

The results show that the extraction solvent influenced the phenolic profile of the date seed colorant. While catechin was efficiently extracted using both water and glycerol–water, the presence of ellagic acid only in the glycerol–water extract suggests that glycerol improved the recovery of less water-soluble phenolic compounds. The slightly higher gallic acid content in the glycerol–water extract further supports the role of solvent composition in phenolic extraction. Since both water and glycerol are food-compatible solvents, the glycerol–water system may be useful for improving the recovery of selected phenolic compounds while maintaining suitability for edible colorant applications.

## 4. Discussion

The preliminary screening experiment demonstrated that the roasting temperature played a critical role in brown color development in date seed extracts. The progressive increase in OD_420_ values observed with increasing roasting temperatures indicates enhanced formation of brown-colored compounds during thermal processing. Such behavior is commonly associated with non-enzymatic browning reactions that occur when food materials are exposed to elevated temperatures [23]. The initiation of brown color development at 90 °C suggests the onset of thermal reactions within the seed matrix, while the substantial increase in OD_420_ values observed between 110 °C and 200 °C indicates accelerated pigment formation at higher temperatures. These changes are likely associated with Maillard reactions and caramelization processes, both of which contribute to the generation of brown-colored compounds during roasting [24]. In Maillard reactions, reducing sugars interact with amino acids to form intermediate compounds that subsequently polymerize into high-molecular-weight brown pigments known as melanoidins [25]. Simultaneously, thermal degradation of carbohydrates may generate additional colored compounds through caramelization pathways [26]. Although the highest OD_420_ value was recorded at 230 °C, the accompanying visual observations revealed excessive darkening and burnt characteristics. This suggests that severe thermal treatment may promote degradation of desirable compounds and formation of burnt products that could negatively affect the quality and acceptability of the resulting colorant [27]. Consequently, temperatures of 90 °C, 110 °C, and 200 °C were selected for further optimization studies as they provided a suitable range for evaluating controlled color development while avoiding excessive thermal degradation. Although the optimized roasting condition (200 °C for 5 min) enhanced brown color development, high-temperature thermal processing may also promote the formation of process contaminants such as acrylamide and furans through Maillard reactions [28]. Recent studies on roasted date seed products have reported that acrylamide levels are generally lower than or comparable to those of conventional roasted coffee and remain below the European benchmark level established for coffee products, indicating no apparent health concern under moderate consumption [29]. However, contaminant formation is influenced by roasting conditions, and further optimization is warranted to balance maximum color development with minimum contaminant formation [29]. Future studies should therefore evaluate acrylamide and furan concentrations under the optimized roasting condition identified in the present study and investigate whether the process can be further refined to minimize contaminant formation while maintaining the desired color intensity. In addition, precursor-reduction approaches such as asparaginase pretreatment and alternative roasting technologies, including vacuum or steam roasting, may be explored to further improve product safety while preserving the color quality and sensory properties of the developed natural colorant [30].The Taguchi optimization successfully identified the processing parameters responsible for maximizing color extraction efficiency from roasted date seed powder. Among the three factors investigated, roasting temperature exerted the greatest influence on OD_420_ values, accounting for 85.75% of the total variation. This dominant contribution indicates that thermal intensity was the primary factor that controls pigment formation during processing. The substantial increase in color intensity observed at higher roasting temperatures further supports the importance of heat-induced chemical transformations in the generation of brown-colored compounds [31]. Elevated temperatures accelerate Maillard reactions, carbohydrate degradation, and the formation of conjugated structures that contribute to brown pigmentation [25]. The increase in OD_420_ values with roasting time suggests that longer exposure to heat allowed these reactions to proceed further, resulting in increased pigment accumulation. However, the comparatively lower contribution of roasting time indicates that temperature was more influential than heating duration. Extraction time exhibited only a negligible effect on OD_420_ values, suggesting that once brown-colored compounds were generated during roasting, they were readily solubilized into the extraction medium. Consequently, extending extraction duration provided limited additional improvement in color recovery. The contour plots further highlighted the interaction between roasting temperature and roasting time, where the highest OD_420_ values were obtained under conditions combining elevated temperatures with longer roasting durations. The validation experiments confirm the reliability of both Taguchi and regression models based on the comparison between experimental and predicted values. The low prediction error values observed during confirmation experiments indicate that the optimization approach was effective in identifying conditions capable of producing highly colored extracts with consistent performance [32]. The optimized roasting condition selected in the present study is also expected to influence the sensory characteristics of the developed colorant. Previous studies have demonstrated that roasting induces the formation of Maillard reaction-derived volatile compounds, including furans and pyrans, which impart characteristic roasted, caramel-like, and coffee-like aroma and flavor notes to date seed products [33]. Metabolomic profiling has further shown that roasted date seeds develop volatile profiles resembling those of roasted coffee, supporting their use as a coffee substitute. Although the flavor and aroma characteristics of the developed date seed colorant were not evaluated in the present study, the product is intended primarily as a natural colorant rather than as a flavoring ingredient and is therefore expected to be incorporated into food formulations at substantially lower concentrations than roasted date seed beverages [29,33]. Consequently, any contribution of roasted flavor or aroma is expected to be considerably lower. At the same time, future studies should evaluate the sensory characteristics of the developed colorant in different food matrices, optimize its inclusion level to minimize any undesirable roasted notes, and assess consumer acceptability to ensure that the desired color can be achieved without adversely affecting flavor or aroma. Although the Taguchi approach successfully optimized extraction parameters and predicted color intensity (OD_420_), detailed time-dependent extraction data required for kinetic modeling were not collected in this study. Future investigations should evaluate extraction kinetics in aqueous and glycerol–water systems to better understand mass transfer behavior and support process scale-up.

The broad absorption band observed around 3310 cm^−1^ is characteristic of O–H stretching vibrations, indicating the presence of hydroxyl-containing compounds within the roasted date seed matrix [34]. Such groups are commonly associated with plant-derived biomolecules and contribute to the chemical complexity of roasted seed materials. The persistence of this absorption band after roasting suggests that oxygen-containing constituents remained present following thermal treatment. The peaks observed at approximately 2915 and 2850 cm^−1^ correspond to aliphatic C–H stretching vibrations and suggest the presence of lipid-derived and carbohydrate-derived structures. The persistence of these peaks indicates that components of these biomolecules remained detectable after roasting. The absorption band at 1740 cm^−1^ is characteristic of carbonyl-containing compounds such as aldehydes, ketones, esters, and lipid oxidation products [35]. The formation of carbonyl-containing compounds is commonly associated with thermal processing and represents an important intermediate stage of non-enzymatic browning reactions, including both caramelization and the Maillard reaction. These carbonyl compounds subsequently participate in condensation and polymerization reactions that contribute to the formation of brown-colored pigments during roasting. Similarly, the absorption band near 1560 cm^−1^ has been associated with aromatic C=C skeletal vibrations and conjugated nitrogen-containing structures commonly reported in thermally processed foods. These structures are consistent with the formation of high-molecular-weight Maillard reaction products (melanoidins), which are considered the principal contributors to the characteristic brown coloration of roasted materials. The peaks detected in the fingerprint region at approximately 1370, 1230, and 1065 cm^−1^ indicate the presence of carbohydrate-associated and oxygen-containing functional groups. These bands suggest that structural modifications of carbohydrate components occurred during roasting while retaining the characteristics of polysaccharide-derived functionalities [17]. The lower-wavenumber bands observed near 880 and 610 cm^−1^ further indicate the presence of aromatic and mineral-associated components within the roasted powder [36]. The simultaneous presence of hydroxyl, carbonyl, aromatic, and carbohydrate-associated functional groups supports the occurrence of extensive chemical transformations during roasting These structural features are characteristic of thermally processed food systems and are consistent with the formation of complex brown polymeric compounds generated through Maillard reactions, caramelization, and limited lipid oxidation. Although FTIR alone cannot unequivocally identify individual melanoidin structures, the observed functional group distribution agrees well with previous reports describing melanoidin-rich roasted products such as coffee, cocoa, and cereal-based materials. Storage conditions significantly influenced the stability of the developed date seed colorant. The progressive increase in OD_420_ values observed during storage indicates that changes continued to occur within the extract after preparation, resulting in the gradual darkening of the colorant over time. The greater increase in OD_420_ values observed in clear bottle samples compared with amber bottle samples highlights the importance of light exposure in determining color stability [37]. Exposure to light can promote oxidation of susceptible compounds present in the extract, leading to the formation of additional colored products and an increase in absorbance [38]. The lower OD_420_ values maintained in amber bottles suggest that protection from light effectively slowed these reactions and improved color retention during storage [39]. Samples stored at room temperature exhibited greater changes in OD_420_ values than refrigerated samples, indicating that higher storage temperatures accelerated degradation and oxidation processes. In contrast, refrigeration slowed these reactions and reduced the rate of color change throughout the storage period [40]. The combined effect of reduced temperature and protection from light was most evident in the refrigerated amber bottle storage condition, which showed the lowest increase in OD_420_ values over 7 days. The findings of the present study demonstrate that appropriate storage conditions provide an effective first strategy for improving the stability of the developed date seed colorant. Among the evaluated conditions, storage in amber bottles under refrigerated conditions resulted in the highest retention of color and bioactive compounds, highlighting the importance of minimizing light exposure and elevated temperatures during storage. For commercial applications, additional formulation strategies may be required to further enhance stability under ambient conditions. These may include the incorporation of suitable food-grade stabilizers, encapsulation technologies, and optimized packaging systems to protect the colorant from oxygen, light, and temperature-induced degradation. Furthermore, long-term storage studies, validation in different food matrices, and process optimization will be essential to establish the robustness and commercial applicability of the developed natural colorant. The gradual decline in TPC and TFC observed during storage indicates that phenolic and flavonoid compounds were susceptible to degradation under the tested storage conditions. The observed 44.6% reduction in TPC and 56.4% reduction in TFC under room-temperature clear bottle storage demonstrates that exposure to light and elevated temperature accelerated the degradation of phenolic and flavonoid compounds. Similar reductions in phenolic and flavonoid contents during storage have been reported for plant-derived extracts and natural colorants, where oxidation and polymerization of phenolic compounds resulted in progressive losses of bioactive constituents [41]. The refrigerated amber bottle treatment retained approximately 92% of the initial TPC and TFC after 7 days, demonstrating that protection from both light and elevated temperature effectively preserved the phytochemical quality of the extract. The simultaneous reduction in TPC, TFC, and increase in OD_420_ values suggest that oxidation and subsequent polymerization of phenolic compounds may have contributed to the formation of darker colored compounds during storage. Such transformations are commonly reported in plant-derived extracts and natural colorant systems [42]. These findings indicate that protection from both light and elevated temperatures is essential for maintaining the phytochemical quality of the developed date seed colorant. The storage stability of the developed colorant could be further improved through the use of oxygen- and light-barrier packaging, refrigeration, and suitable food-grade stabilizers or encapsulation techniques. These approaches may help minimize oxidative degradation and extend shelf life and therefore warrant further investigation before commercial application.

The decline in ABTS and DPPH antioxidant activities during storage reflects the progressive loss of antioxidant constituents within the date seed colorant extract. Since phenolic compounds, flavonoids, tannins, and other reducing molecules contribute substantially to the antioxidant activity of plant-derived extracts, the reductions observed in both assays are consistent with the decreases in TPC and TFC [43]. The similar trends observed for both ABTS and DPPH assays further confirm that storage-related losses in antioxidant activity were associated with degradation of bioactive phytochemicals within the extract. These findings highlight the importance of appropriate storage conditions for preserving the functional properties of the developed date seed colorant and support the use of refrigerated amber bottles to maximize antioxidant stability during storage [44].

The gradual increase in pH observed during storage indicates progressive chemical changes within the date seed colorant extract. This increase may be associated with a reduction in acidic constituents and the transformation of organic compounds during storage [45]. Similar increases in pH have been reported in fruit- and plant-based products, where storage-related biochemical reactions are accompanied by a decrease in titratable acidity and modifications in the chemical composition of the matrix [46]. In the present study, the pH changes occurred concurrently with reductions in phenolic content, flavonoid content, tannin content, and antioxidant activity, suggesting an overall decline in bioactive compound stability during storage. The concurrent increase in pH together with the decline in total phenolic and flavonoid contents suggests that oxidative degradation and polymerization reactions likely contributed to the observed biochemical and physicochemical changes during storage. Phenolic compounds are susceptible to oxidation, forming reactive quinone intermediates that subsequently undergo condensation and polymerization reactions to generate higher-molecular-weight brown polymers with reduced antioxidant activity and lower reactivity toward the Folin–Ciocalteu reagent. Similarly, flavonoids may undergo oxidation, structural rearrangement, and degradation during storage, contributing to the observed reduction in total flavonoid content. The progressive depletion of these acidic phenolic constituents, together with their conversion into higher-molecular-weight polymeric products, may also contribute to the gradual increase in pH, while exposure to oxygen, light, and elevated temperature is likely to accelerate these degradation processes, particularly under room-temperature storage conditions. These degradation reactions may alter the balance between acidic and non-acidic constituents, thereby contributing to the gradual increase in pH observed during storage [47]. Notably, samples stored in amber bottles in refrigerated conditions exhibited the smallest pH variations throughout the storage period, indicating better preservation of extract composition and supporting the superior storage stability observed for this condition.

Since the developed colorant is intended for food applications, its performance under different food pH conditions is an important consideration. The present study evaluated the physicochemical stability of the colorant under its native extraction conditions but did not investigate color stability across the wider pH range encountered in different food systems. Existing studies on date seed-derived systems have demonstrated that pH significantly influences their stability and functionality, with greater stability observed under mildly acidic conditions (pH 6–7) and reduced stability at lower pH values [47]. Therefore, future studies should evaluate the stability of the developed date seed colorant in representative food matrices, including acidic beverages, dairy products, and bakery formulations, and develop appropriate formulation strategies to maintain color quality under varying pH, processing, and storage conditions. Unlike phenolic compounds, flavonoids, and antioxidant constituents, minerals are inorganic elements and are, therefore, inherently more stable during storage. This explains the minimal variation observed in the concentrations of most macro- and micronutrients throughout the study period [48]. The stability of potassium, calcium, phosphorus, magnesium, sodium, zinc, copper, and manganese indicates that storage temperature and packaging conditions had little influence on the mineral composition of the developed colorant. The slight reduction in iron concentration observed in some treatments may be associated with changes in extract chemistry during storage [48]. As pH increased over time, soluble iron may have undergone oxidation and subsequent complexation or precipitation, reducing the concentration of dissolved iron detected in the extract [49]. This observation is consistent with the gradual increase in pH reported during storage. The high mineral stability observed under all storage conditions suggests that the developed colorant can retain its mineral composition even when changes occur in phenolic content and antioxidant activity.

The HPLC analysis confirmed the presence of phenolic compounds in the developed date seed colorant and demonstrated that extraction solvent influenced the recovery of individual phenolic constituents. Catechin was efficiently extracted by both solvent systems, indicating its relatively high polarity and solubility in aqueous media. The comparable concentrations observed in both extracts suggest that water alone was sufficient for recovering this compound. In contrast, differences were observed for gallic acid and ellagic acid. The slightly higher gallic acid concentration detected in the glycerol–water extract indicates that the addition of glycerol enhanced its extraction efficiency [50]. Previous studies have shown that glycerol can form stronger hydrogen-bond interactions with gallic acid than water or other alcohol-based solvents, thereby improving its solvation and recovery. The presence of three hydroxyl groups in glycerol increases the solvent’s ability to interact with phenolic acids, resulting in enhanced extraction efficiency [50]. More notably, ellagic acid was detected exclusively in the glycerol–water extract, suggesting that solvent composition played an important role in its recovery. This behavior can be associated with the physicochemical properties of glycerol, which, owing to its multiple hydroxyl groups, enhances hydrogen-bonding interactions and modifies the polarity of the extraction medium. These interactions improve the solvation of relatively less water-soluble phenolic compounds, such as ellagic acid, and may also facilitate the disruption of phenolic–cell wall interactions, thereby promoting the release of bound phenolics into the extraction solvent. This observation is consistent with the relatively lower aqueous solubility of ellagic acid compared with catechin and gallic acid, making its extraction more dependent on solvent composition [50]. Catechin, gallic acid, and ellagic acid are well-known antioxidant phenolics, and their detection supports the antioxidant activity observed in the developed colorant [51]. The results indicate that the incorporation of glycerol into the extraction medium can modify the phenolic profile of the extract and improve the recovery of selected compounds while maintaining the use of food-compatible solvents. This may be advantageous for developing natural colorants with enhanced functional properties and potential applications in sustainable food systems.

The findings of the present study demonstrate the technical feasibility of producing a natural brown colorant from date seed waste using a simple roasting and aqueous extraction process. However, successful industrial implementation will require further optimization of process efficiency, extraction yield, and formulation strategies to improve storage stability, together with large-scale production to ensure economic viability. Although the present study demonstrated the initial physicochemical stability of the developed date seed colorant, comprehensive shelf-life evaluation, including microbiological assessment, dry matter determination, and long-term stability under different storage conditions and in various food matrices, is required before commercial application. Future studies should therefore focus on techno-economic feasibility assessment, process scale-up, development of improved stabilization strategies such as protective packaging and encapsulation, validation in different food matrices, sensory evaluation, food safety assessment, and regulatory compliance. Addressing these aspects will facilitate the translation of the developed natural colorant from laboratory-scale research to commercially viable clean-label food applications.

## 5. Conclusions

This study successfully developed a sustainable natural brown colorant from date seed waste using Taguchi optimization. The optimum processing conditions of 200 °C roasting temperature, 5 min roasting time, and 15 min extraction time produced the highest color intensity, while validation experiments confirmed the reliability of the optimization approach. Refrigerated storage in amber bottles provided the highest retention of color quality, phenolic compounds, flavonoids, antioxidant activity, and physicochemical stability, whereas the mineral composition remained largely unaffected during storage. FTIR and HPLC analyses confirmed the presence of bioactive functional groups and phenolic compounds associated with the color-forming and antioxidant properties of the developed extract.

These findings demonstrate the potential of date seed waste as a sustainable source of natural brown colorants for clean-label food applications while supporting waste valorization and circular economy principles. The optimized process provides a scientific foundation for the future development of commercially viable natural food colorants from date seed waste. However, further studies are required before commercial application to evaluate the formation of process contaminants under the optimized roasting conditions, assess sensory properties and color stability in different food matrices with varying pH, and investigate formulation and stabilization strategies, including protective packaging and encapsulation, to improve shelf life. Future research should also focus on long-term storage stability, techno-economic feasibility, food safety, and process scale-up to support industrial production of date seed-derived natural colorants.

## Figures and Tables

**Table 1:** Processing parameters, symbols, and levels used in the Taguchi L9 orthogonal array design for optimization of date seed colorant extraction.

Parameter	Symbol	Level 1	Level 2	Level 3
Roasting temperature (°C)	T	90	110	200
Roasting time (min)	Rt	1	3	5
Extraction time (min)	Et	5	10	15

**Table 2:** Experimental combinations generated using the Taguchi L9 orthogonal array design.

Run	Roasting Temperature (T)	Roasting Time (Rt)	Extraction Time (Et)
1	90	1	5
2	90	3	10
3	90	5	15
4	110	1	10
5	110	3	15
6	110	5	5
7	200	1	15
8	200	3	5
9	200	5	10

**Table 3:** Preliminary screening study showing the effect of roasting temperature on brown color development and absorbance (OD_420_) of date seed extracts.

Temperature (°C)	OD420	Observation
70	0.12	Very light brown color
90	0.35	Initiation of brown color formation
110	0.51	Moderate brown coloration
150	0.74	Increased brown intensity
200	0.96	Strong brown color development
230	1.21	Excessive darkening and burnt characteristics

**Table 4:** Taguchi L9 experimental design with OD_420_ response and corresponding S/N ratio values for date seed colorant extraction.

	Roasting Temperature (T)	Roasting Time (Rt)	Extraction Time (Et)	Response	S/N Ratio
1	90	1	5	0.44	−7.13095
2	90	3	10	0.525	−5.59681
3	90	5	15	0.58	−4.73144
4	110	1	10	0.525	−5.59681
5	110	3	15	0.645	−3.80881
6	110	5	5	0.67	−3.4785
7	200	1	15	0.775	−2.21397
8	200	3	5	0.875	−1.15984
9	200	5	10	0.915	−0.77158

**Table 5:** Response table for S/N ratios and parameter ranking.

Level	T	Rt	Et
1	−5.820	−4.981	−3.923
2	−4.295	−3.522	−3.988
3	−1.382	−2.994	−3.585
Delta	4.438	1.987	0.404
Rank	1	2	3

**Table 6:** Comparison of experimental OD_420_ values with Taguchi and regression model predictions along with percentage error values for date seed colorant extraction.

Run	Roasting Temp	Roasting Time	Extraction Time	Response	Taguchi Prediction	Error %	Regression Prediction	Error %
1	90	1	5	0.44	0.434	1.26	0.459	−4.37
2	90	3	10	0.525	0.529	−0.85	0.533	−1.44
3	90	5	15	0.58	0.581	−0.19	0.606	−4.46
4	110	1	10	0.525	0.526	−0.21	0.519	1.23
5	110	3	15	0.645	0.639	0.86	0.592	8.23
6	110	5	5	0.67	0.674	−0.66	0.665	0.71
7	200	1	15	0.775	0.779	−0.57	0.791	−2.01
8	200	3	5	0.875	0.881	−0.70	0.856	2.13
9	200	5	10	0.915	0.909	0.61	0.930	−1.61
T-200, Rt-5, Et-15	200	5	15	0.927	0.921	0.64	0.932	−0.56

**Table 7:** Mineral composition (mg/100 g DW) of date seed colorant extracts during storage under different conditions.

Condition	Day	K	Ca	P	Mg	Na	Fe	Zn	Cu	Mn
RT clear	0	112.4 (±8.7)	31.02 (±1.2)	27.3 (±1.4)	17.3 (±1.2)	9.1 (±1.1)	2.9 (±0.5)	0.4 (±0.1)	0.18 (±0.01)	0.1 (±0.01)
	2	118.5 (±9.2)	33.01 (±1.1)	26.7 (±0.9)	17.1 (±0.9)	9.9 (±1.7)	2.7 (±0.6)	0.44 (±0.1)	0.12 (±0.01)	0.08 (±0.005)
	5	115.3 (±7.2)	31.72 (±1.2)	27.9 (±0.7)	17.8 (±1.1)	9.3 (±0.9)	2.2 (±0.3)	0.47 (±0.2)	0.15 (±0.01)	0.09 (±0.004)
	7	116.6 (±8.7)	31.52 (±0.9)	27.11 (±0.7)	18.1 (±1.5)	9.1 (±0.9)	2.1 (±0.5)	0.49 (±0.2)	0.11 (±0.01)	0.09 (±0.005)
RT amber	0	112.4 (±8.7)	31.02 (±1.2)	27.3 (±1.4)	17.3 (±1.2)	9.1 (±1.1)	2.9 (±0.5)	0.4 (±0.1)	0.18 (±0.01)	0.1 (±0.01)
	2	114.4 (±6.6)	32.54 (±1.5)	28.2 (±1.7)	16.9 (±0.8)	8.8 (±0.7)	2.9 (±0.8)	0.39 (±0.3)	0.14 (±0.01)	0.1 (±0.009)
	5	117.2 (±4.2)	32.16 (±1.1)	28.5 (±1.4)	17.8 (±1.6)	9.2 (±0.8)	2.5 (±0.4)	0.47 (±0.2)	0.11 (±0.01)	0.09 (±0.005)
	7	113.2 (±8.8)	31.79 (±1.3)	27.7 (±1.9)	18.1 (±0.9)	9.5 (±1.1)	2.5 (±0.7)	0.42 (±0.1)	0.10 (±0.01)	0.09 (±0.005)
5 °C clear	0	112.4 (±8.7)	31.02 (±1.2)	27.3 (±1.4)	17.3 (±1.2)	9.1 (±1.1)	2.9 (±0.5)	0.4 (±0.1)	0.18 (±0.01)	0.1 (±0.01)
	2	118.4 (±7.7)	33.08 (±0.9)	26.9 (±1.6)	16.9 (±0.8)	9.7 (±1.4)	2.8 (±0.8)	0.47 (±0.2)	0.13 (±0.01)	0.08 (±0.007)
	5	114.8 (±7.9)	32.7 (±1.1)	27.9 (±1.3)	17.7 (±1.9)	9.9 (±1.9)	2.5 (±0.6)	0.45 (±0.2)	0.15 (±0.01)	0.09 (±0.008)
	7	117.3 (±9.1)	31.9 (±1.2)	26.8 (±0.9)	17.3 (±0.8)	9.7 (±1.4)	2.4 (±0.8)	0.42 (±0.3)	0.14 (±0.01)	0.1 (±0.009)
5 °C amber	0	112.4 (±8.7)	31.02 (±1.2)	27.3 (±1.4)	17.3 (±1.2)	9.1 (±1.1)	2.9 (±0.5)	0.4 (±0.1)	0.18 (±0.01)	0.1 (±0.01)
	2	109.2 (±10.3)	31.9 (±1.3)	27.77 (±1.8)	17.2 (±1.1)	9.3 (±0.9)	3.1 (±0.9)	0.45 (±0.3)	0.11 (±0.01)	0.008 (±0.006)
	5	117.5 (±6.6)	33.1 (±1.1)	28.1 (±1.5)	17.4 (±1.3)	9.5 (±1.3)	2.7 (±0.6)	0.41 (±0.1)	0.12 (±0.01)	0.1 (±0.01)
	7	115 (±7.3)	30.9 (±0.9)	27.7 (±1.4)	17.8 (±1.1)	9.2 (±1.1)	2.6 (±0.5)	0.46 (±0.3)	0.16 (±0.01)	0.09 (±0.008)

**Table 8:** HPLC phenolic profile of date seed colorant extracts prepared using aqueous and glycerol–water extraction solvents.

Extraction Solvent	Catechin (µg/g DW)	Ellagic Acid (µg/g DW)	Gallic Acid (µg/g DW)
Aqueous extract	11.35 (±0.9)	ND	4.5 (±0.5)
Glycerol + water	11.10 (±1.1)	7.8 (±0.3)	5.25 (±0.8)

## Data Availability

The original contributions presented in this study are included in the article. Further inquiries can be directed to the corresponding authors.

## Abbreviations

L9	Taguchi Orthogonal Array (9 experimental runs)
OD_420_	Optical Density at 420 nm (Absorbance at 420 nm)
FTIR	Fourier Transform Infrared Spectroscopy
GAE	Gallic Acid Equivalents
DW	Dry Weight
QE	Quercetin Equivalents
HPLC	High-Performance Liquid Chromatography
UAE	United Arab Emirates
UV–Vis	Ultraviolet–Visible spectroscopy
T	Roasting Temperature
Rt	Roasting Time
Et	Extraction time
S/N ratio	Signal-to-Noise ratio
ANOVA	Analysis of Variance
ATR	Attenuated Total Reflectance
TPC	Total Phenolic Content
TFC	Total Flavonoid Content
DPPH	2,2-diphenyl-1-picrylhydrazyl radical
ABTS	2,2′-azino-bis(3-ethylbenzothiazoline-6-sulfonic acid)
TEAC	Trolox Equivalent Antioxidant Capacity
SE	Standard Error
ICP-MS	Inductively Coupled Plasma Mass Spectrometry
Min	Minute
C	Carbon
O	Oxygen
H	Hydrogen
pH	Power of Hydrogen
K	Potassium
Ca	Calcium
P	Phosphorus
Mg	Magnesium
Na	Sodium
Fe	Iron
Zn	Zinc
Cu	Copper
Mn	Manganese
